# Comparison of two genetic strategies for diagnostic work-up of hypertrophic cardiomyopathy: impact on the diagnosis of Fabry disease or transthyretin amyloidosis

**DOI:** 10.1186/s13023-025-03815-z

**Published:** 2025-06-10

**Authors:** Aurélien Palmyre, Fairouz Koraichi, Flavie Ader, Erwan Donal, Céline Bordet, Pascal de Groote, Laurence Faivre, Patricia Reant, Annick Toutain, Karine Nguyen, Bertrand Isidor, Anne-Claire Brehin, Lise Legrand, Estelle Gandjbakhch, Julie Proukhnitzky, Richard Isnard, Nicolas Mansencal, Jean-François Pruny, Jean-Pierre Rabes, Bruno Francou, Catherine Caillaud, Pascale Richard, Philippe Charron

**Affiliations:** 1 APHP, Service de Génétique, Hôpital Ambroise Paré, Boulogne-Billancourt, France; 2 APHP, Département de Génétique & ICAN Institute, Hôpital Pitié-Salpêtrière, Paris, France; 3 INSERM, UMRS 1166 and ICAN Institute for Cardiometabolism and Nutrition, Sorbonne Université, 75013 Paris, France; 4 APHP, UF Cardiogénétique et Myogenetique moleculaire et cellulaire, Service de Biochimie Métabolique, Hôpital Pitié-Salpêtrière, 75013 Paris, France; 5 UFR de Pharmacie, Université Paris Cité, 4 av de l’observatoire, 75006 Paris, France; 6 Département cardiologie / service de genetique clinique, CHU Rennes Hôpital de Pontchaillou, Rennes, France; 7 Service de Cardiologie, CHU Lille, 59000 Lille, France; 8 Inserm U1167, Institut Pasteur de Lille, 59000 Lille, France; 9 Service de Génétique Médicale, FHU TRANSLAD, CHU Dijon Bourgogne, Dijon, France; 10 University Hospital Centre of Bordeaux, Bordeaux University, Bordeaux, France; 11 UF de genetique medicale, CHU de Tours Hôpital Bretonneau, Tours, France; 12 Département de Génétique Médicale, CHU de Marseille, Hôpital d’enfants de la Timone, Marseille, France; 13 Service de genetique medicale, Hôpital mère et enfant CHU de Nantes, CHU Nantes, Nantes, France; 14 Inserm U1245 and CHU Rouen, Department of Pathology, Department of Genetics and Reference Center for Developmental Abnormalities, Univ Rouen Normandie, Normandie Univ, 76000 Rouen, France; 15 APHP, Département de Cardiologie, Hôpital Pitié-Salpêtrière, Paris, France; 16 APHP, Service de Cardiologie, Hôpital Ambroise Paré, Boulogne-Billancourt, France; 17 APHP, Laboratoire de Biochimie et Génétique Moléculaire, hopital Ambroise Paré (APHP. Université Paris-Saclay), Boulogne-Billancourt & UFR Simone Veil-Santé, UVSQ, Montigny-Le-Bretonneux, France; 18 APHP, Hôpital Bicêtre, Génétique Pharmacogénétique et Hormonologie, Hôpital Bicêtre, Université Paris-Sud, Le Kremlin Bicêtre, France; 19 Département de biochimie, metabolique, proteomique, hopital Necker Enfants Malades, Paris, France

**Keywords:** Fabry disease, Transthyretin amyloidosis, Hypertrophic cardiomyopathy, NGS, Sanger sequencing, Diagnosis

## Abstract

**Background:**

Diagnostic work-up of patients with hypertrophic cardiomyopathy is crucial for appropriate management. However, the optimal genetic strategy remains debatable. We compared two strategies: targeted testing based on careful examination of clinical red flags versus large multigene panel analysis without gene prioritization. We applied the strategy to the diagnosis of Fabry disease or Hereditary Transthyretin Amyloidosis (*GLA* or *TTR* genes respectively).

**Results:**

We studied 341 hypertrophic cardiomyopathy index patients. Patients of subgroup 1 (n = 42) had careful clinical analysis and high suspicion of Hereditary Transthyretin Amyloidosis or Fabry disease. They underwent targeted Sanger sequencing. Patients in subgroup 2 (n = 299) did not have clinical selection, and underwent next-generation sequencing analysis of 107 cardiac genes.

The yield of genetic testing for pathogenic/likely pathogenic variants in *GLA* and/or *TTR* was 28.6% in subgroup 1 (12/42: 5 *TTR* and 7 *GLA*) versus 1.0% in subgroup 2 (3/299: 1 *TTR* and 2 *GLA*), *p* < 0.01. Genetic results were obtained after a median of 26.0 days [IQR = 18–59.8] in subgroup 1 versus 193.5 days [IQR = 174–218] in subgroup 2, *p* < 0.01. Finally, genetic testing cost was 615.60€ or 769.50€ for *TTR* or *GLA* targeted analysis respectively, versus 1503.90€ for multigene panel analysis.

**Conclusions:**

Both molecular strategies in hypertrophic cardiomyopathy patients are useful for the identification of pathogenic/likely pathogenic variants in *TTR/GLA* genes. However, targeted genetic testing based on clinical red flags identified causal mutations more efficiently, faster and at a lower cost. Careful clinical analysis is therefore important in guiding molecular strategy and may reduce diagnostic wandering and accelerate delivery of appropriate therapy.

**Supplementary Information:**

The online version contains supplementary material available at 10.1186/s13023-025-03815-z.

## Introduction

Hypertrophic cardiomyopathy (HCM) is defined by the presence of segmental or diffuse left ventricular hypertrophy (LVH) of the wall thickness that is not solely explained by abnormal loading conditions. Appropriate diagnostic work-up of patients with HCM is a major step in identifying the precise underlying aetiology, progress towards precision medicine and delivery of dedicated therapies when available. Fabry disease and hereditary transthyretin cardiac amyloidosis (ATTR) are examples of rare diseases with wandering and delay in diagnosis, but with a major impact of the diagnosis since specific therapies are effective in reducing cardiovascular morbidity and mortality [1–3].

However, the optimal strategy for genetic testing remains debatable. With the advancement of next-generation sequencing (NGS), it is possible to sequence simultaneously all the genes known to be involved in HCM, including *GLA* gene and the *TTR* gene involved in Fabry disease and ATTR amyloidosis, respectively. However targeted sequencing strategies based on the presence of red flags may have advantages [4, 5] since the sequencing step is simpler and quicker, although data on the comparative evaluation of the two strategies are lacking in the field of cardiomyopathies.

The aim of this study was to compare two sequencing strategies: targeted testing based on careful examination of clinical red flags versus large multigene panel analysis without gene prioritization. To do so, we compared the efficiency and cost-effectiveness of these two diagnostic methods, using the examples of Fabry disease and hereditary ATTR.

## Methods

### Patients

We collected data from 341 index patients with HCM/LVH (unexplained left ventricular hypertrophy), from ten centres in France including two referral centres with a strategy of clinical red flags analysis and eight centres without clinical selection. The criterion used for wall thickness was ≥ 15 mm, or ≥ 12 mm in the presence of associated red flags and suspicion of a particular aetiology such as ATTR amyloidosis [6] or Fabry disease [2, 7]. Subgroup 1 consisted of patients with careful clinical analysis and high suspicion of ATTR or Fabry disease based on red flags (Supplementary Table 1). They underwent targeted sequencing. Subgroup 2 consisted of patients without clinical selection based on potential red flags. They directly underwent NGS multigene panel analysis. The study protocol conforms to the ethical guidelines of the 1975 Declaration of Helsinki, written informed consent was obtained from each patient, and the study complied with the reference methodology (MR004) of the French Data Protection Authority (CNIL).

### Genetic analysis

Patients' DNAs were extracted from peripheral blood and sequencing methods were used as described in Supplementary section (Supplementary Methods), either through NGS technology performed to screen 107 genes involved in cardiac diseases (Supplementary Table 2) or a targeted gene Sanger sequencing performed for *GLA* or *TTR* genes.

We evaluated each variant according to current American College of Medical Genetics and Genomics guidelines [8] that recommend classifying variants into five categories: pathogenic, likely pathogenic, uncertain significance, likely benign, or benign. Only pathogenic and likely pathogenic variant were considered (Supplementary Table 3).

### Evaluation of the time required for each analysis

We defined the time required for each analysis as the time between the date of reception of the samples by the lab and the date of validation of the results by the medical technologist in charge of the analysis as reported in the official genetic results.

### Comparative analysis

The two strategies were compared regarding efficiency (yield of pathogenic/likely pathogenic (P/LP) variants) and the time required to obtain the genetic results (from date of sampling to date of laboratory report). The cost of the analyses was fixed to their respective French prices (RIHN nomenclature, “Référentiel des actes de biologie médicale et d’anatomocytopathologie Innovants Hors Nomenclature”).

### Statistical analysis

Fisher’s exact test was used to compare the identification rate between NGS analysis and the targeted strategy, while the Mann–Whitney–Wilcoxon test was applied to compare the time required to obtain the results. *p*-values lower than 0.05 were considered as indicating statistical significance. R software (v 1.4.1103) was used for statistical computations.

## Results

We enrolled 341 HCM patients including 42 patients in subgroup 1 (red flag analysis and suspicion of ATTR or Fabry disease) and 299 patients in subgroup 2 (no detailed clinical selection).

**Fig. 1:**
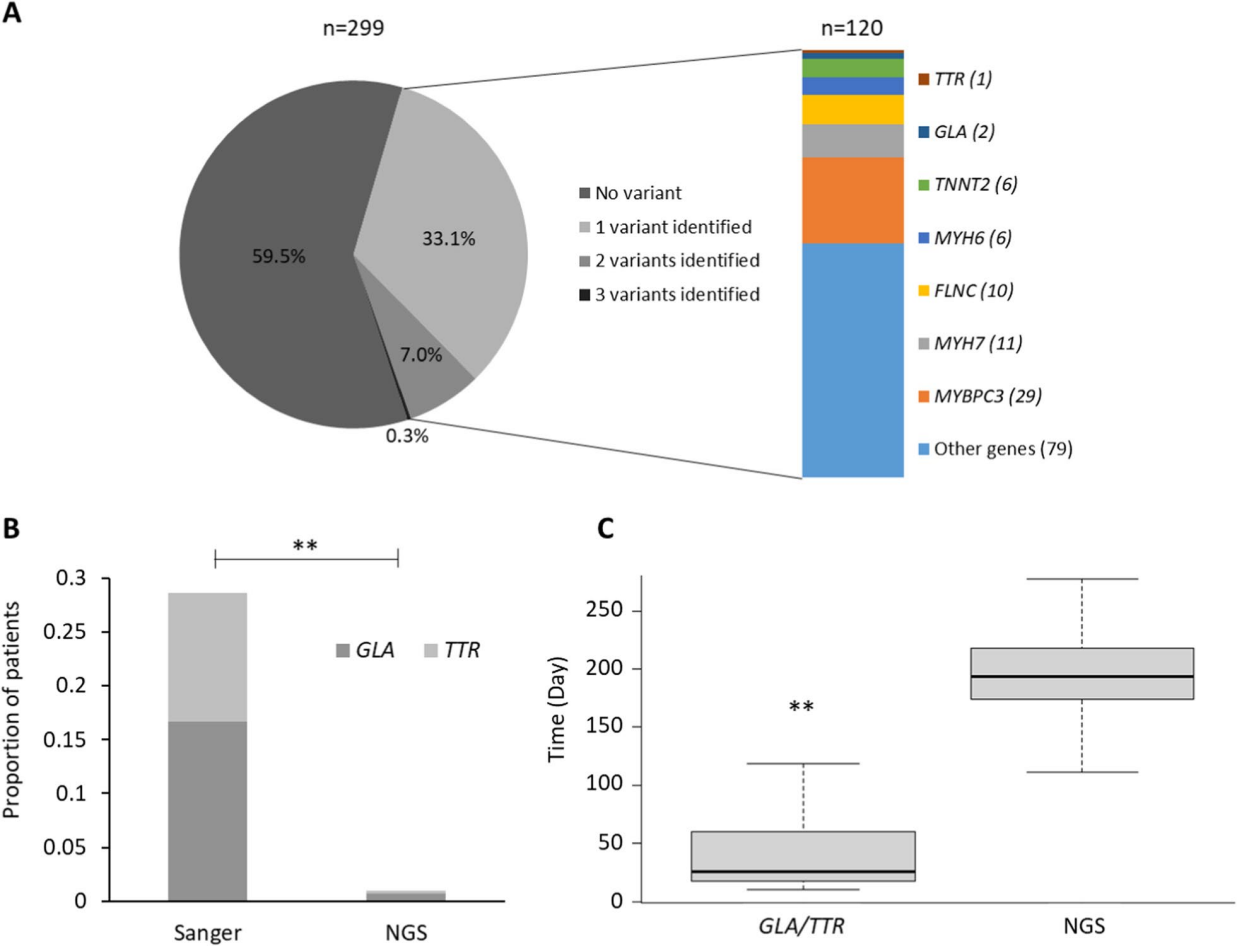
Outcomes of multigene analysis versus targeted analysis. **A** Pathogenic or likely pathogenic (P/LP) variants identified with NGS technology in the population with hypertrophic cardiomyopathy. The population of 299 patients with confirmed hypertrophic cardiomyopathy was screened for 107 genes with NGS technology. We identified 179 patients who had no P/LP variants. Among the 120 patients who carried at least one P/LP variant, 144 variants were identified: 98 patients carried 1 variant, 21 patients carried 2 variants and 1 patient carried 3 variants. **B** Proportion of patients carrying *GLA* and *TTR* P/LP variants as determined by Sanger sequencing or the NGS strategy (**p < 0.01). **C** Boxplot of the time to receipt of the result of NGS multi-gene analysis and Sanger analysis of the *GLA* and *TTR* genes (**p < 0.01)

Genetic variant screening in subgroup 2 of 299 HCM patients (82 women and 217 men; age at diagnosis 40.8 ± 18.2 years) using NGS strategy allowed identification of 120 patients (40%) with at least one P/LP variant (Fig. 1A). Among them, one patient had a variant in the *TTR* gene (p.(Val142Ile)) and two woman have a variant in the *GLA* gene (p.(Val269Ala), p.(Ala143Thr)) (Table 1, S2) (mean age for these three patients: 63.7 ± 12.0 years). In subgroup 1 of 42 patients (13 women and 29 men; age at diagnosis 60.3 ± 18.9 years), Sanger sequencing identified 7 patients with a *GLA* P/LP variant (one with p.Ile317Thr, one with p.(Trp226*) and five with p.(Asn215Ser)) and 5 with a *TTR* P/LP variant (4 with p.(Val142Ile) also known as Val122Ile and one with p.(Val30Met)), all heterozygous (Table 1, Supplementary Table 2). Hence, targeted analysis of *GLA* and/or *TTR* performed better in mutation identification than NGS analysis (12/42 = 28.6% vs. 3/299 = 1.0%; *p* < 0.01) (Fig. 1B).

**Table 1:** Patients carrying *GLA* or *TTR* P/LP variant identified by Sanger sequencing or NGS

	ID	Sex	Gene	DNA Sequence	Deduced Amino Acid Change	Age At Diagnosis of LVH/HCM (year)	Ethnicity	Conduction defect (including bundle branch blocks)	Maximum Left Ventricular Thickness (mm)	Left Ventricular Ejection Fraction	Cardiac MRI	Extra-cardiac Manifestations	Positive Technetium-99 m Pyrophosphate Scintigraphy	Reduced Alpha-Gal A Enzyme Activity
Sanger Squencing Analysis	1	F	*GLA*	c.950 T > C	p.(Ile317Thr)	53	Other	Short PR	21	70%	Infero-lateral left ventricular LGE	Cerebrovascular accident	–	Yes
	2	M	*GLA*	c.678 G > A	p.(Trp226*)	31	North Africa	–	13	60%	Decreased T1	Cornea verticillata, hearing loss, angiokeratoma, renal failure neuropathic pain	–	Yes
	3	M	*GLA*	c.644 A > G	p.(Asn215Ser)	61	Caucasian	–	18	74%	Infero-lateral left ventricular LGE	–	–	Yes
	4	M	*GLA*	c.644 A > G	p.(Asn215Ser)	19	Caucasian	–	23	–	–	–	–	Yes
	5	M	*GLA*	c.644 A > G	p.(Asn215Ser)	20	Caucasian	–	18	74%	Infero-lateral left ventricular LGE	–	–	Yes
	6	M	*GLA*	c.644 A > G	p.(Asn215Ser)	48	Caucasian	AVB3	13,1	51,50%	Infero-lateral left ventricular LGE	Proteinuria	–	Yes
	7	M	*GLA*	c.644 A > G	p.(Asn215Ser)	53	Caucasian	bundle branch blocks	18	60%	Infero-lateral left ventricular LGE	Proteinuria	–	Yes
	8	M	*TTR*	c.424G > A	p.(Val142Ile) (Val122Ile)	76	North Africa	bundle branch blocks	17	30%	Left and right ventricular LGE and atrials LGE	Cerebrovascular accident	Yes	–
	9	M	*TTR*	c.424G > A	p.(Val142Ile) (Val122Ile)	74	South Africa	AVB1	14,3	42%	Left ventricular myocardial annular cockade LGE	Carpal tunnel syndrome	Yes	–
	10	M	*TTR*	c.424G > A	p.(Val142Ile) (Val122Ile)	62	Other	AVB2	16	39%	Subendocardial diffuse LGE	–	Yes	No
	11	M	*TTR*	c.424G > A	p.(Val142Ile) (Val122Ile)	83	South Africa	AVB	20	38%	–	Sensorimotor neuropathy carpal tunnel syndrome	–	No
	12	F	*TTR*	c.148G > A	p.(Val50Met)	40	Caucasian	–	20	71%	–	Liver transplantation, Neuropathy	No	–
NGS	13	F	*GLA*	c.427G > A	p.(Ala143Thr)	63	Caucasian	–	19	55–60%	–	–	–	–
	14	F	*GLA*	c.806 T > C	p.(Val269Ala)	52	Caucasian	–	17	72%	–	–	No	No
	15	M	*TTR*	c.424G > A	p.(Val142Ile) (Val122Ile)	76	South Africa	–	23	30%	–	Renal failure	–	–

*AVB* atrioventricular block, *LGE* late gadolinium enhancement, *LVH* left ventricular hypertrophy, *HCM* hypertrophic cardiomyopathy

The median time required to obtain the results by NGS panel analysis was 193.5 days [IQR = 174–218]. This is significantly longer (*p* < 0.01) than the time required to obtain the results by targeted analysis: 26.0 days [IQR = 18–59.8] (Fig. 1C).

According to the French nomenclature, the cost of a single genetic test was fixed at 1503.90€ for the NGS-based multi-gene panel analysis versus 615.60€ for *TTR* Sanger sequencing and 769.50€ for *GLA* Sanger sequencing.

## Discussion

Appropriate aetiological diagnosis of HCM, and reduction in the time of the process, is key issue, especially for some diseases such as ATTR amyloidosis or Fabry disease, where specific therapies are recommended (Tafamidis for ATTR or enzyme replacement therapies or chaperone therapy in Fabry disease). Moreover, it has been shown that late diagnosis of such diseases, and delayed therapy, is significantly associated with a lower efficiency of therapies and increased cardiovascular mortality [9, 10]. In addition, early diagnosis leads to early appropriate screening of relatives and therefore early management of the whole family.

To our knowledge, ours is the first comparative evaluation of two sequencing strategies for diagnostic work-up of patients with HCM. We compared a large multigene panel analysis using NGS in patients without detailed clinical analysis and without gene prioritization versus targeted testing using Sanger sequencing based on careful examination of clinical red flags. We observed that both molecular strategies are useful for the identification of P/LP variants in *TTR* and *GLA* genes. However, targeted genetic testing based on clinical red flags identified causal mutations more efficiently (P/LP yield 28.6% vs 1.0%), faster (median 26 days vs 193) and at lower cost (~ half the cost) as compared to NGS multi-gene panel analysis. Interestingly, the prevalence of 1% of *GLA*/*TTR* pathogenic variants with multigene panel was very similar to a previous French cohort with exome sequencing [11].

Although the NGS multigene panel strategy has become the standard for molecular diagnosis in HCM, our results suggest that there is still a role for targeted gene sequencing based on careful clinical analysis. Both strategies have pro and contra. The NGS strategy allows parallel analysis of most genes involved in HCM and therefore considers all potential aetiologies, and may be helpful for identifying unsuspected rare causes, especially in centres with limited clinical expertise. Conversely, it has been shown that increasing the number of genes by NGS did not increase the diagnostic yield in HCM, but expanded panels increased the yield of variants of unknown significance [12] which lead to extensive additional bioinformatics work and delayed receipt of the definitive molecular results (3–9 months for the standard routine in most academic laboratories). Therefore, our results suggest that targeted genetic analysis should be performed first in the presence of clinical red flag, whereas multigene analysis should be performed first in the other cases, or secondary when no mutation is identified by the previous strategy. For these two diseases, if they are highly suspected based on the clinical diagnostic and no genetic variants are identified, we suggest performing whole genome sequencing, which allows for better coverage of deep intronic regions and potentially of other genes that may affect the expression of the *GLA* or *TTR* genes.

Our study may have some limitations. The targeted sequencing method requires detailed clinical analysis and expertise to suspect potential underlying causes, but knowledge is rapidly progressing in this regard. Targeted sequencing was performed with Sanger sequencing, which is performed less and less, but restricted NGS panels are increasingly used and are of similar utility. The time to results for both strategies, and related costs, vary greatly according to countries and academic versus private companies, but we think that our results apply to a majority of situations and countries. Finally, the targeted sequencing method can only be applied to rare diseases with few causative disease genes, otherwise, the analysis of several genes involved in the rare disease will increase the total cost of the strategy.

## Conclusion

The NGS multigene panel strategy and the targeted sequencing strategy are both useful tools for detecting genetic variants for Fabry disease and hereditary transthyretin amyloidosis in patient presenting with HCM. However, targeted genetic testing based on clinical red flags identified causal mutations more efficiently, faster and at lower cost. Careful clinical analysis therefore appears important in guiding molecular strategy and may reduce diagnostic wandering and accelerate delivery of appropriate therapy.

## Supplementary Information

Supplementary Material 1.

## Data Availability

The datasets generated during and/or analysed during the current study are not publicly available due to the sensitivity of personal data processed but are available from the corresponding author on reasonable request.

## Abbreviations

ATTR: Hereditary transthyretin amyloidosis
HCM: Hypertrophic cardiomyopathy
LVH: Left ventricular hypertrophy
NGS: Next-generation sequencing
P/LP variant: Pathogenic/likely pathogenic variant
