# Supplementary material for: Comparison of two genetic strategies for diagnostic work-up of hypertrophic cardiomyopathy: impact on the diagnosis of Fabry disease or transthyretin amyloidosis
Source: Orphanet J Rare Dis. 2025 Jun 10;20:294. doi: 10.1186/s13023-025-03815-z (PMC12153184; doi:10.1186/s13023-025-03815-z)
Supplement: Supplementary file 1 — Supplementary Material 1. [file 13023_2025_3815_MOESM1_ESM.docx]

**Supplementary information of “Comparison of two genetic strategies for diagnostic work-up of hypertrophic cardiomyopathy: impact on the diagnosis of Fabry Disease or Transthyretin Amyloidosis”**

Supplementary information

1. **Supplementary Methods**
2. **Supplementary Tables**
   1. Supplementary Table 1
   2. Supplementary Table 2
   3. Supplementary Table 3
3. **Supplementary References**
4. **Supplementary Methods**

Patients' DNAs were extracted from peripheral blood with Qiasymphony (Qiagen, Hilden, Germany) and qualitatively checked using Tape Station DNA genomic array (Agilent, Santa Clara, California). Custom targeted gene enrichment and DNA library preparation were performed using the NimbleGen EZ choice probes and Kappa Library preparation kit according to the manufacturer's instructions (Nimblegen, Roche Diagnostics, Madison, Wisconsin).The targeted regions were sequenced using the Illumina MiSeq platform on a 500 cycle Flow Cell (Illumina, Santa Cruz, California) and MiSeq Software generates FASTQ format files after demultiplexing patients' sequences. Merged single reads and paired-end reads were then aligned on Hg19 human reference genome, using Burrows-Wheeler Aligner BWA-MEM. Variant calling was performed using the Genome Analysis Tool Kit Haplotype Caller program then annotated using ANNOVAR. The NGS technology was used to screen 107 genes (1810 exons/533600 base pairs) involved in cardiac diseases (Supplementary Table 2).

A targeted gene strategy through Sanger sequencing was performed for *GLA* and *TTR* genes, from DNA isolated from peripheral blood. Mutation numbering was based on HGVS nomenclature (NCBI Reference Sequence: NM_000169.3 and NM_000371.4).

**2. Supplementary tables**

**2.1 Supplementary Table 1** “Red flags” manifestations of Fabry disease and amyloidosis adapted from Hung *et al*., Gertz *et al*., Hoffmann *et al*. and Oerlemans *et al.*[1–3].

|  | | **Fabry Disease** | **Amyloidosis** |
| --- | --- | --- | --- |
| **Cardiovascular manifestations** | **ECG**  **Echocardiography**  **CMR imaging**  **Other** | Short PQ interval  Impaired variability of cardiac frequency  Reduced global longitudinal strain, especially in infero-lateral segment  Mild-to-moderate aortic root dilation  Mitral and aortic valve thickening with mild-to-moderate regurgitation  Low native T1  Mid-layer posterolateral late gadolinium enhancement  Angina pectoris, myocardial infarction | Congestive heart failure (including shortness of breath, generalized fatigue, and peripheral oedema)  Conduction blocks  Low QRS voltage or disproportionally low voltage in the presence of increased left ventricular wall thickness/LVH  Pseudo-infarct pattern in the absence of wall motion abnormalities on echocardiography  Symmetrical LVH (and RVH) in the absence of aortic stenosis or longstanding hypertension  Preserved LVEF, but reduced global longitudinal strain with apical sparing  Transmural or subendocardial LGE not related to a coronary artery territory, diffuse atrial LGE, RV LGE, suboptimal nulling  Increased native T1 values, increased extracellular volume, myocardial oedema (T2)  Disproportionally high level of NT-proBNP, chronically elevated troponin at low level with normal CAG |
| **Dermatological manifestation** | | Angiokeratoma  Hypohidrosis/anhidrosis  Hyperhidrosis  Lymphoedema | Sweating abnormalities |
| **Peripheral nervous system** | | Neuropathic pain | Progressive symmetric peripheral sensorimotor neuropathy |
| **Gastrointestinal manifestations** | | Abdominal pain  Diarrhoea  Constipation  delayed intestinal passage | Nausea and vomiting  Early satiety  Diarrhoea/constipation  Unintentional weight loss |
| **Eyes** | | Cornea verticillata  Tortuositas vasorum  Uveitis  Conjunctival aneurysms | Dark floaters  Glaucoma  Abnormal blood vessels in eye  Pupillary abnormalities |
| **Ears** | | Acute/chronic hearing loss  Tinnitus  Dizziness | Hearing loss |
| **Nephropathy** | | Proteinuria  Renal failure  Albuminuria | Proteinuria  Renal failure  Albuminuria  Mild azotaemia |
| **Central nervous system** | | TIA/stroke  White matter lesions | TIA/stroke |
| **Others** | |  | Bilateral carpal tunnel syndrome  Lumbar spinal stenosis  Spontaneous distal biceps tendon rupture  Orthostatic hypotension  Erectile dysfunction  Recurrent urinary tract infection (due to urinary retention) |
| **Family history** | | Cardiomyopathy  Renal failure  Stroke | Cardiomyopathy  Renal failure  Stroke |

**2.2 Supplementary Table 2** List of the 107 genes involved in cardiac diseases and analysed with NGS technology

| **Disease** | **Genes** | **Ref Seq** | **Chr** | **5'end** | **3'end** | **Coding exons** |
| --- | --- | --- | --- | --- | --- | --- |
| **CARDIOMYOPATHY** | *AARS2* | NM_020745,2 | chr6 | 44268000 | 44281162 | 22 |
| **CARDIOMYOPATHY** | *ABCC9* | NM_020297 | chr12 | 21950324 | 22089628 | 38 |
| **CARDIOMYOPATHY** | *ACAD9* | NM_014049 | chr3 | 128603445 | 128631501 | 18 |
| **CARDIOMYOPATHY** | *ACTA1* | NM_001100_ | chr1 | 229567195 | 229569893 | 6 |
| **CARDIOMYOPATHY** | *ACTC1* | [NM_005159.4](http://www.ncbi.nlm.nih.gov/entrez/query.fcgi?cmd=Search&db=Nucleotide&term=NM_005159&doptcmdl=GenBank&tool=genome.ucsc.edu) | chr15 | 35082524 | 35087087 | 6 |
| **CARDIOMYOPATHY** | *ACTN2* | [NM_001103.2](http://www.ncbi.nlm.nih.gov/entrez/query.fcgi?cmd=Search&db=Nucleotide&term=NM_001103&doptcmdl=GenBank&tool=genome.ucsc.edu) | chr1 | 236849900 | 236926700 | 21 |
| **CARDIOMYOPATHY** | *AGK* | NM_018238,3 | chr7 | 141254955 | 141353884 | 15 |
| **CARDIOMYOPATHY** | *ANKRD1* | [NM_014391.2](http://www.ncbi.nlm.nih.gov/entrez/query.fcgi?cmd=Search&db=Nucleotide&term=NM_014391&doptcmdl=GenBank&tool=genome.ucsc.edu) | chr10 | 92672442 | 92681119 | 9 |
| **CARDIOMYOPATHY** | *BAG3* | [NM_004281.3](http://www.ncbi.nlm.nih.gov/entrez/query.fcgi?cmd=Search&db=Nucleotide&term=NM_004281&doptcmdl=GenBank&tool=genome.ucsc.edu) | chr10 | 121410839 | 121437129 | 4 |
| **CARDIOMYOPATHY** | *C2orf64 (COA5)* | [NM_001008215.1](http://www.ncbi.nlm.nih.gov/entrez/query.fcgi?cmd=Search&db=Nucleotide&term=NM_001008215&doptcmdl=GenBank&tool=genome.ucsc.edu) | chr2 | 99216947 | 99225156 | 3 |
| **CARDIOMYOPATHY** | *CALR3* | NM_145046.3 | chr19 | 16589868 | 16607003 | 9 |
| **CARDIOMYOPATHY** | *COX10* | NM_001303,3 | chr17 | 13973399 | 14111797 | 7 |
| **CARDIOMYOPATHY** | *COX15* | NM_078470,4 | chr10 | 101474382 | 101492172 | 9 |
| **CARDIOMYOPATHY** | *CSRP3* | NM_003476.3 | chr11 | 19203577 | 19232118 | 5 |
| **CARDIOMYOPATHY** | *DES* | [NM_001927.3](http://www.ncbi.nlm.nih.gov/entrez/query.fcgi?cmd=Search&db=Nucleotide&term=NM_001927&doptcmdl=GenBank&tool=genome.ucsc.edu) | chr2 | 220282977 | 220290839 | 9 |
| **CARDIOMYOPATHY** | *DTNA* | NM_001390.4 | chr18 | 32335940 | 32471808 | 21 |
| **CARDIOMYOPATHY** | *EMD* | [NM_000117.2](http://www.ncbi.nlm.nih.gov/entrez/query.fcgi?cmd=Search&db=Nucleotide&term=NM_000117&doptcmdl=GenBank&tool=genome.ucsc.edu) | chrX | 153607727 | 153609706 | 6 |
| **CARDIOMYOPATHY** | *EYA4* | NM_004100.4 | chr6 | 133562495 | 133853258 | 19 |
| **CARDIOMYOPATHY** | *FHL1* | NM_001159702 | chrX | 135229559 | 135293518 | 6 |
| **CARDIOMYOPATHY** | *FLNC* | [NM_001458.4](http://www.ncbi.nlm.nih.gov/entrez/query.fcgi?cmd=Search&db=Nucleotide&term=NM_001458&doptcmdl=GenBank&tool=genome.ucsc.edu) | chr7 | 128470509 | 128498874 | 48 |
| **CARDIOMYOPATHY** | *GLA* | [NM_000169.3](http://www.ncbi.nlm.nih.gov/entrez/query.fcgi?cmd=Search&db=Nucleotide&term=NM_000169&doptcmdl=GenBank&tool=genome.ucsc.edu) | chrX | 100652716 | 100663014 | 7 |
| **CARDIOMYOPATHY** | *HEY2* | NM_012259 | chr6 | 126070873 | 126080998 | 5 |
| **CARDIOMYOPATHY** | *JPH2* | NM_020433.4 | chr20 | 42740337 | 42816218 | 5 |
| **CARDIOMYOPATHY** | *LAMP2* | [NM_002294.2](http://www.ncbi.nlm.nih.gov/entrez/query.fcgi?cmd=Search&db=Nucleotide&term=NM_002294&doptcmdl=GenBank&tool=genome.ucsc.edu) | chrX | 119564925 | 119603519 | 9 |
| **CARDIOMYOPATHY** | *LDB3 (ZASP)* | [NM_007078.2](http://www.ncbi.nlm.nih.gov/entrez/query.fcgi?cmd=Search&db=Nucleotide&term=NM_007078&doptcmdl=GenBank&tool=genome.ucsc.edu) | chr10 | 88428064 | 88493126 | 13 |
| **CARDIOMYOPATHY** | *LMNA* | [NM_170707.2](http://www.ncbi.nlm.nih.gov/entrez/query.fcgi?cmd=Search&db=Nucleotide&term=NM_170707&doptcmdl=GenBank&tool=genome.ucsc.edu) | chr1 | 156084211 | 156109122 | 12 |
| **CARDIOMYOPATHY** | *MRPL44* | NM_022915,3 | chr2 | [224822121](http://genome.ucsc.edu/cgi-bin/hgc?hgsid=329452229&g=htcCdnaAli&i=NM_022915&c=chr2&l=224820120&r=224834430&o=224822120&aliTable=refSeqAli&table=refGene) | 224832431 | 4 |
| **CARDIOMYOPATHY** | *MYBPC3* | [NM_000256.3](http://www.ncbi.nlm.nih.gov/entrez/query.fcgi?cmd=Search&db=Nucleotide&term=NM_000256&doptcmdl=GenBank&tool=genome.ucsc.edu) | chr11 | 47353172 | 47374477 | 34 |
| **CARDIOMYOPATHY** | *MYH6* | NM_002471.3 | chr14 | 23851199 | 23877486 | 37 |
| **CARDIOMYOPATHY** | *MYH7 (B MHC)* | NM_000257.2 | chr14 | 23881887 | 23903141 | 38 |
| **CARDIOMYOPATHY** | *MYL2* | [NM_000432.3](http://www.ncbi.nlm.nih.gov/entrez/query.fcgi?cmd=Search&db=Nucleotide&term=NM_000432&doptcmdl=GenBank&tool=genome.ucsc.edu) | chr12 | 111348763 | 111358463 | 7 |
| **CARDIOMYOPATHY** | *MYL3* | [NM_000258.2](http://www.ncbi.nlm.nih.gov/entrez/query.fcgi?cmd=Search&db=Nucleotide&term=NM_000258&doptcmdl=GenBank&tool=genome.ucsc.edu) | chr3 | 46899716 | 46904958 | 6 |
| **CARDIOMYOPATHY** | *MYLK2* | NM_033118.3 | chr20 | 30407178 | 30422500 | 12 |
| **CARDIOMYOPATHY** | *MYOM1* | NM_003803.3 | chr18 | 3066805 | 3220106 | 37 |
| **CARDIOMYOPATHY** | *MYOZ2* | [NM_016599.3](http://www.ncbi.nlm.nih.gov/entrez/query.fcgi?cmd=Search&db=Nucleotide&term=NM_016599&doptcmdl=GenBank&tool=genome.ucsc.edu) | chr4 | 120057000 | 120108000 | 5 |
| **CARDIOMYOPATHY** | *MYPN* | [NM_032578.2](http://www.ncbi.nlm.nih.gov/entrez/query.fcgi?cmd=Search&db=Nucleotide&term=NM_032578&doptcmdl=GenBank&tool=genome.ucsc.edu) | chr10 | 69549958 | 69640893 | 19 |
| **CARDIOMYOPATHY** | *NEBL* | NM_006393.2 | chr10 | 21068903 | 21186531 | 28 |
| **CARDIOMYOPATHY** | *NEXN* | [NM_144573.3](http://www.ncbi.nlm.nih.gov/entrez/query.fcgi?cmd=Search&db=Nucleotide&term=NM_144573&doptcmdl=GenBank&tool=genome.ucsc.edu) | chr1 | 78153507 | 78181932 | 12 |
| **CARDIOMYOPATHY** | *NKX2-5* | [NM_004387.3](http://www.ncbi.nlm.nih.gov/entrez/query.fcgi?cmd=Search&db=Nucleotide&term=NM_004387&doptcmdl=GenBank&tool=genome.ucsc.edu) | chr5 | 172659365 | 172662212 | 2 |
| **CARDIOMYOPATHY** | *PDLIM3* | NM_014476.4 | chr4 | 186421815 | 186456712 | 8 |
| **CARDIOMYOPATHY** | *PLN* | NM_002667.3 | chr6 | 118869380 | 118881908 | 1 |
| **CARDIOMYOPATHY** | *PRDM16* | NM_022114_ | chr1 | 2985774 | 3350425 | 17 |
| **CARDIOMYOPATHY** | *PRKAG2* | [NM_016203.3](http://www.ncbi.nlm.nih.gov/entrez/query.fcgi?cmd=Search&db=Nucleotide&term=NM_016203&doptcmdl=GenBank&tool=genome.ucsc.edu) | chr7 | 151251200 | 151575543 | 16 |
| **CARDIOMYOPATHY** | *PSEN1* | NM_000021.3 | chr14 | 73603143 | 73690399 | 10 |
| **CARDIOMYOPATHY** | *PSEN2* | NM_000447.2 | chr1 | 227058273 | 227083804 | 10 |
| **CARDIOMYOPATHY** | *RBM20* | [NM_001134363.1](http://www.ncbi.nlm.nih.gov/entrez/query.fcgi?cmd=Search&db=Nucleotide&term=NM_001134363&doptcmdl=GenBank&tool=genome.ucsc.edu) | chr10 | 112404154 | 112598198 | 14 |
| **CARDIOMYOPATHY** | *SCO2* | [NM_001169109.1](http://www.ncbi.nlm.nih.gov/entrez/query.fcgi?cmd=Search&db=Nucleotide&term=NM_001169109&doptcmdl=GenBank&tool=genome.ucsc.edu) | chr22 | 50962016 | 50962869 | 1 |
| **CARDIOMYOPATHY** | *SDHA* | NM_004168,2 | chr5 | 218353 | 256895 | 15 |
| **CARDIOMYOPATHY** | *SGCD1* | NM_000337.5 | chr5 | 155753767 | 156194798 | 8 |
| **CARDIOMYOPATHY** | *SLC25A4 (ANT1)* | NM_001151,3 | chr4 | 186064474 | 186068209 | 4 |
| **CARDIOMYOPATHY** | *SYNPO2* | [NM_133477.2](http://www.ncbi.nlm.nih.gov/entrez/query.fcgi?cmd=Search&db=Nucleotide&term=NM_133477&doptcmdl=GenBank&tool=genome.ucsc.edu) | chr4 | 119808000 | 119960000 | 5 |
| **CARDIOMYOPATHY** | *TAZ* | [NM_000116.3](http://www.ncbi.nlm.nih.gov/entrez/query.fcgi?cmd=Search&db=Nucleotide&term=NM_000116&doptcmdl=GenBank&tool=genome.ucsc.edu) | chrX | 153639925 | 153649530 | 11 |
| **CARDIOMYOPATHY** | *TCAP* | [NM_003673.3](http://www.ncbi.nlm.nih.gov/entrez/query.fcgi?cmd=Search&db=Nucleotide&term=NM_003673&doptcmdl=GenBank&tool=genome.ucsc.edu) | chr17 | 37821516 | 37822450 | 2 |
| **CARDIOMYOPATHY** | *TMEM70* | [NM_017866.5](http://www.ncbi.nlm.nih.gov/entrez/query.fcgi?cmd=Search&db=Nucleotide&term=NM_017866&doptcmdl=GenBank&tool=genome.ucsc.edu) | chr8 | 74888146 | 74894048 | 3 |
| **CARDIOMYOPATHY** | *TMPO* | NM_003276.2 | chr12 | 98909351 | 98929412 | 4 |
| **CARDIOMYOPATHY** | *TNNC1* | [NM_003280.2](http://www.ncbi.nlm.nih.gov/entrez/query.fcgi?cmd=Search&db=Nucleotide&term=NM_003280&doptcmdl=GenBank&tool=genome.ucsc.edu) | chr3 | 52485259 | 52488077 | 6 |
| **CARDIOMYOPATHY** | *TNNI3* | [NM_000363.4](http://www.ncbi.nlm.nih.gov/entrez/query.fcgi?cmd=Search&db=Nucleotide&term=NM_000363&doptcmdl=GenBank&tool=genome.ucsc.edu) | chr19 | 55663104 | 55669083 | 8 |
| **CARDIOMYOPATHY** | *TNNT2* | [NM_001001430.1](http://www.ncbi.nlm.nih.gov/entrez/query.fcgi?cmd=Search&db=Nucleotide&term=NM_000364&doptcmdl=GenBank&tool=genome.ucsc.edu) | chr1 | 201328136 | 201346828 | 15 |
| **CARDIOMYOPATHY** | *TPM1* | [NM_001018005.1](http://www.ncbi.nlm.nih.gov/entrez/query.fcgi?cmd=Search&db=Nucleotide&term=NM_001018005&doptcmdl=GenBank&tool=genome.ucsc.edu) | chr15 | 63334757 | 63358273 | 10 |
| **CARDIOMYOPATHY** | *TTN* | NM_001256850,1 | chr2 | 179390717 | 179672150 | 312 |
| **CARDIOMYOPATHY** | *TTR* | [NM_000371.](http://www.ncbi.nlm.nih.gov/entrez/query.fcgi?cmd=Search&db=Nucleotide&term=NM_000371&doptcmdl=GenBank&tool=genome.ucsc.edu)4 | chr18 | 29171745 | 29178781 | 4 |
| **CARDIOMYOPATHY** | *VCL* | NM_014000.2 | chr10 | 75757872 | 75879914 | 22 |
| **MARFAN** | *FBN1* | NM_000138,4 | chr15 | 48700503 | 48937985 | 65 |
| **NOONAN et al.** | *KRAS* | NM_004985,3 | chr12 | 25358180 | 25403854 | 4 |
| **NOONAN et al.** | *PTPN11* | NM_002834,3 | chr12 | 112856536 | 112947717 | 15 |
| **NOONAN et al.** | *RAF1* | NM_002880,3 | chr3 | 12625100 | 12705700 | 16 |
| **NOONAN et al.** | *SOS1* | NM_005633,3 | chr2 | 39208690 | 39347604 | 23 |
| **POMPE** | *GAA* | [NM_000152.3](http://www.ncbi.nlm.nih.gov/entrez/query.fcgi?cmd=Search&db=Nucleotide&term=NM_000152&doptcmdl=GenBank&tool=genome.ucsc.edu) | chr17 | 78078157 | 78093247 | 19 |
| **ARVC** | *CASQ2* | NM_001232,3 | chr1 | 116242626 | 116311426 | 11 |
| **ARVC** | *CTNNA3* | NM_013266.2 | chr10 | 67679725 | 69455949 | 17 |
| **ARVC** | *DSC2* | NM_024422,3 | chr18 | 28645942 | 28682388 | 16 |
| **ARVC** | *DSG2* | NM_001943,3 | chr18 | 29078027 | 29128814 | 15 |
| **ARVC** | *DSP* | NM_004415,2 | chr6 | 7541870 | 7586946 | 24 |
| **ARVC** | *JUP* | NM_002230,2 | chr17 | 39910859 | 39942964 | 13 |
| **ARVC** | *PKP2* | NM_004572,3 | chr12 | 32943680 | 33049780 | 14 |
| **ARVC** | *RYR2* | NM_001035,2 | chr1 | 237205702 | 237997288 | 105 |
| **ARVC** | *TMEM43* | NM_024334,2 | chr3 | 14166440 | 14185180 | 12 |
| **ATRIAL FIBRILLATION** | *GJA5* | NM_005266,5 | chr1 | 147228332 | 147245484 | 1 |
| **ATRIAL FIBRILLATION** | *KCNA5* | NM_002234,2 | chr12 | 5153085 | 5155954 | 1 |
| **ATRIAL FIBRILLATION** | *NPPA* | NM_006172 | chr1 | 11905767 | 11907840 | 3 |
| **BRUGADA SYNDROME** | *CACNA2D1* | NM_000722,2 | chr7 | 81579418 | 82073031 | 39 |
| **BRUGADA SYNDROME** | *CACNB2* | NM_201596,2 | chr10 | 18429606 | 18830688 | 14 |
| **BRUGADA SYNDROME** | *GPD1L* | MN_015141,3 | chr3 | 32148003 | 32210207 | 8 |
| **BRUGADA SYNDROME** | *SCN1B* | NM_199037,3 | chr19 | 35521592 | 35525174 | 3 |
| **BRUGADA SYNDROME** | *SCN5A* | NM_198056,2 | chr3 | 38589553 | 38674850 | 27 |
| **Long QT Syndrome** | *AKAP9* | [NM_005751.4](http://www.ncbi.nlm.nih.gov/entrez/query.fcgi?cmd=Search&db=Nucleotide&term=NM_005751&doptcmdl=GenBank&tool=genome.ucsc.edu) | chr7 | 91570189 | 91739897 | 50 |
| **Long QT Syndrome** | *ANK2* | [NM_001148.4](http://www.ncbi.nlm.nih.gov/entrez/query.fcgi?cmd=Search&db=Nucleotide&term=NM_001148&doptcmdl=GenBank&tool=genome.ucsc.edu) | chr4 | 113970785 | 114304896 | 46 |
| **Long QT Syndrome** | *CACNA1C* | NM199460,2 | chr12 | 2162416 | 2807115 | 50 |
| **Long QT Syndrome** | *CAV3* | MN033337,2 | chr3 | 8775486 | 8788451 | 2 |
| **Long QT Syndrome** | *HCN4* | NM_005477,2 | chr15 | 73612200 | 73661605 | 8 |
| **Long QT Syndrome** | *KCND3* | NM_004980,4 | chr1 | 112318454 | 112531777 | 7 |
| **Long QT Syndrome** | *KCNE1* | [NM_000219.3](http://www.ncbi.nlm.nih.gov/entrez/query.fcgi?cmd=Search&db=Nucleotide&term=NM_000219&doptcmdl=GenBank&tool=genome.ucsc.edu) | chr21 | 35820779 | 35822554 | 1 |
| **Long QT Syndrome** | *KCNE1L* | NM_012282,2 | chrX | 108866729 | 108868764 | 1 |
| **Long QT Syndrome** | *KCNE2* | [NM_172201.1](http://www.ncbi.nlm.nih.gov/entrez/query.fcgi?cmd=Search&db=Nucleotide&term=NM_172201&doptcmdl=GenBank&tool=genome.ucsc.edu) | chr21 | 35742683 | 35743491 | 1 |
| **Long QT Syndrome** | *KCNH2* | NM000238,2 | chr7 | 150642044 | 150675402 | 15 |
| **Long QT Syndrome** | *KCNJ2* | NM000891,2 | chr17 | 68165676 | 68176183 | 1 |
| **Long QT Syndrome** | *KCNJ5* | NM_000890,3 | chr11 | 128761313 | 128787951 | 2 |
| **Long QT Syndrome** | *KCNJ8* | NM_004982,2 | chr12 | 21917889 | 21927747 | 2 |
| **Long QT Syndrome** | *KCNQ1* | NM 000218,2 | chr11 | 2466221 | 2870340 | 16 |
| **Long QT Syndrome** | *RANGRF* | NM_016492,4 | chr17 | 8191969 | 8193409 | 5 |
| **Long QT Syndrome** | *SCN2B* | NM_004588,4 | chr11 | 118033519 | 118047337 | 4 |
| **Long QT Syndrome** | *SCN3B* | NM018400,3 | chr11 | 123499895 | 123525315 | 5 |
| **Long QT Syndrome** | *SCN4B* | NM174934,3 | chr11 | 118004092 | 118023630 | 5 |
| **Long QT Syndrome** | *SNTA1* | NM_003098,2 | chr20 | 31996158 | 32027853 | 8 |
| **Short QT Syndrome** | *CACNA1B* | NM_000718,2 | chr9 | 140772241 | 141019076 | 47 |
| **ARRHYTHMIA** | *KCNE3* | [NM_005472.4](http://www.ncbi.nlm.nih.gov/entrez/query.fcgi?cmd=Search&db=Nucleotide&term=NM_005472&doptcmdl=GenBank&tool=genome.ucsc.edu) | chr11 | 74167988 | 74168841 | 1 |
| **ARRHYTHMIA** | *TGFB3* | NM_003239,2 | chr14 | 76424442 | 76448092 | 7 |

**2.3 Supplementary Table 3** Classification of *GLA* and *TTR* variants retained

Interpretation of variant was performed according to current American College of Medical Genetics and Genomics guidelines[4].

*Interpretated as such, especially considering ACMG criteria, but considered disputable by some authors.

SIFT score (SIFTori). Scores range from 0 to 1. The smaller the score the more likely the SNP has a damaging effect.

Revel score range from 0 to 1. The larger the score the more likely the SNP has a damaging effect.

PhyloP100way score: the greater the score, the more conserved the site.

^#^This variant in GLA gene is a nonsense variant. Revel score and SIFT score are not applicable for a nonsense variant [5]. Moreover, this variant has not been found in the control population of gnomAD. Considering all these arguments, according to the ACMG recommendations [4], this variant is classified as likely pathogenic.

| **ID** | **Sex** | **Gene** | **DNA Sequence** | **Deduced**  **Amino Acid**  **Change** | **Sequence Variant**  **Nomenclature** | **GnomAD**  **frequency** | **GnomAD**  **allel count** | **SIFT** | **REVEL** | **Conservation**  **PhyloP100way** | **Publication** | **Conclusion** |
| --- | --- | --- | --- | --- | --- | --- | --- | --- | --- | --- | --- | --- |
| **1** | F | *GLA* | c.950 T>C | p.(Ile317Thr) | NM_000169.3 | 0 | 0 | 0 | 0.953 | 9.312 | yes | pathogenic |
| **2** | M | *GLA* | c.678 G>A | p.(Trp226Ter) | NM_000169.3 | 0 | 0 | NA^#^ | NA^#^ | 7.840 | no | likely pathogenic |
| **3-4-5-6-7** | M | *GLA* | c.644 A>G | p.(Asn215Ser) | NM_000169.3 | 0.00000545 | 1/83422 | 0.004 | 0.72 | 8.017 | yes | pathogenic |
| **8-9-10-11-15** | M | *TTR* | c.424G>A | p.(Val142Ile) (Val122Ile) | NM_000371.4 | 0.00113 | 283/251408 | 0.023 | 0.645 | 3.219 | yes | pathogenic |
| **12** | F | *TTR* | c.148G>A | p.(Val50Met | NM_000371.4 | 0 | 0 | 0.287 | 0.53 | 1.488 | yes | likely pathogenic |
| **13** | F | *GLA* | c.427G>A | p.(Ala143Thr) | NM_000169.3 | 0.000545 | 100/183467 | 0.004 | 0.858 | 6.115 | yes | likely pathogenic* |
| **14** | F | *GLA* | c.806 T>C | p.(Val269Ala) | NM_000169.3 | 0 | 0 | 0.002 | 0.949 | 9.325 | yes | pathogenic |

1. **Supplementary References**

1. Gertz MA, Mauermann ML, Grogan M, Coelho T. Advances in the treatment of hereditary transthyretin amyloidosis: A review. Brain Behav. 2019;9(9):1–12.

2. Hung CL, Wu YW, Lin CC, Lai CH, Juang JJM, Chao TH, et al. 2021 Tsoc Expert Consensus on the Clinical Features, Diagnosis, and Clinical Management of Cardiac Manifestations of Fabry Disease. Acta Cardiol Sin. 2021;37(4):337–54.

3. Oerlemans MIFJ, Rutten KHG, Minnema MC, Raymakers RAP, Asselbergs FW, de Jonge N. Cardiac amyloidosis: the need for early diagnosis. Netherlands Hear J [Internet]. 2019 Nov 29;27(11):525–36. Available from: http://link.springer.com/10.1007/s12471-019-1299-1

4. Richards S, Aziz N, Bale S, Bick D, Das S, Gastier-Foster J, et al. Standards and guidelines for the interpretation of sequence variants: A joint consensus recommendation of the American College of Medical Genetics and Genomics and the Association for Molecular Pathology. Genet Med. 2015;17(5):405–24.

5. Ioannidis NM, Rothstein JH, Pejaver V, Middha S, McDonnell SK, Baheti S, et al. REVEL: An Ensemble Method for Predicting the Pathogenicity of Rare Missense Variants. Am J Hum Genet [Internet]. 2016;99(4):877–85. Available from: http://dx.doi.org/10.1016/j.ajhg.2016.08.016
